# Supplementary material for: Subjective experiences of cognitive decline and receiving a diagnosis of dementia: qualitative interviews with people recently diagnosed in memory clinics in the UK
Source: BMJ Open. 2019 Aug 1;9(8):e026071. doi: 10.1136/bmjopen-2018-026071 (PMC6688685; doi:10.1136/bmjopen-2018-026071)
Supplement: Supplementary data [file bmjopen-2018-026071supp001.pdf]

Supplementary Files

Supplementary table 1: Participants' diagnosis/dementia type description[1, 2].

Alzheimer's	The common cause of dementia, described by memory loss and other cognitive abilities serious enough to interfere with daily life.
Vascular dementia	Caused by cerebrovascular disease, where blood vessels in the brain are damaged and brain tissue injured, depriving brain cells of vital oxygen and nutrients.
Mixed dementia	Abnormalities characteristic of more than one type of dementia occur simultaneously in the brain. In the most common form abnormal protein deposits associated with Alzheimer's disease coexist with blood vessel problems linked to vascular dementia.
Lewy body dementia	Caused by abnormal microscopic deposits that damage brain cells over time. Leads to a decline in thinking, reasoning and independent function.
Parkinson's dementia	A decline in thinking and reasoning that develops in many people living with Parkinson's at least a year after diagnosis.<br>Brain changes caused by Parkinson's, often affect mental functions, including memory and the ability to pay attention, make sound judgments and plan the steps needed to complete a task.
Semantic dementia	A variant of Frontotemporal dementia (progressive nerve cell loss in the brain's frontal lobes or its temporal lobes), where individuals lose the ability to understand or formulate words in a spoken sentence.

[1] Descriptions of participants' diagnoses - table 2.
[2] Adapted from: Alzheimer's Association: Types of Dementia, https://www.alz.org/alzheimers-dementia/what-is-dementia/types-of-dementia (accessed 08.03.2019).

Supplementary table 2: Cognitive tests

Mini Mental State Exam (MMSE) (Arevalo-Rodriguez et al 2015)	• 10–12mins to administer and score<br>• Cut-off for dementia: 23/24 out of 30<br>• Cognitive domains: attention and orientation, memory, registration, recall, calculation, language and ability to draw a complex polygon
Addenbrooke's Cognitive Examination III (ACE-III) (Noone, 2015)	• 15–20mins to administer and score<br>• Cut-off for dementia: 82–88/100<br>• Cognitive domains: Attention; Memory; Verbal fluency; Language; and Visuospatial abilities
MiniACE (Hsieh et al 2015)	• 5mins to administer and score<br>• Cut-off for dementia: 21/25 out of 30<br>• Cognitive domains: orientation, memory, language and visuospatial function
